# Energetic Cost of Statistical Order-Degree Change in a Fermions’ Set

**DOI:** 10.3390/e24060752

**Published:** 2022-05-26

**Authors:** Flavia Pennini, Angelo Plastino, Gustavo Luis Ferri, Angel Ricardo Plastino

**Affiliations:** 1 Departamento de Física, Universidad Católica del Norte, Av. Angamos 0610, Antofagasta 3580000, Chile; 2 Departamento de Física, Facultad de Ingenieria, Universidad Nacional de Mar del Plata (UNMDP), CONICET, Mar del Plata 7600, Argentina; 3 Instituto de Física La Plata–CCT-CONICET, Universidad Nacional de La Plata, C.C. 727, La Plata 1900, Argentina; plastino@fisica.unlp.edu.ar; 4 Departamento de Física, Universidad Nacional de La Pampa, Santa Rosa 6300, Argentina; glferri2002@yahoo.com; 5 CeBio-Departamento de Ciencias Básicas, Universidad Nacional del Noroeste de la Prov. de Buenos Aires (UNNOBA), CONICET, Junin 6000, Argentina; arplastino@unnoba.edu.ar

**Keywords:** statistical mechanics’ quantifiers, order-disorder disjunction, fermions

## Abstract

We discuss novel many-fermions thermodynamics’ features. They refer to the energy cost associated to order-disorder changes. Our thermal quantum statistical scenario is controlled by suitable fermion-fermion interactions. We deal with two well-known quantum interactions that operate within an exactly solvable model. This model is able to adequately describe some aspects of fermion-dynamics, particularly level-crossings. We describe things via employment of Gibbs’ canonical ensemble strictures. We show that judicious manipulation of the energy cost associated to statistical order (disorder) variations generates useful information-quantifiers. The underlying idea is that changes in the degree of order are intimately linked to level-crossings energetic costs.

## 1. Introduction

This work explores new features in statistical mechanics. We will discuss novel many-fermions’ thermodynamics features. They refer to the energy cost associated to order-disorder changes whenever our thermal quantum statistical scenario is controlled by suitable fermion-fermion interactions. Let us start with the essential notion of entropy.

Entropy is intimately linked with the idea of disorder, as it is well known. Its order-counterpart (OC) has taken many forms in the literature, but here we will concentrate our attention on a special OC-notion called disequilibrium *D* (see Ref. [1] and references therein). This quantity *D* is the Euclidean distance in probability space between the actual probability distribution and the uniform one. Since the uniform distribution can be thought of as the most ”disordered one”, the larger *D* the larger the order-degree.

Based on the quantity *D* we will try to establish novel links between the notions of entropy, negentropy (simply −S, the negative of the entropy), statistical order, and free energy, in the context of the thermal description of many fermion systems. We will appeal to simple exactly solvable fermion models, that are particularly valuable in testing new notions referring to the intricacies of the quantum many body problem, without appealing to huge hamiltonian matrices [2]. *It is widely accepted that the best way to understand a topic is first to grasp specific, well-chosen cases and then to worry afterward about how to generalize from this understanding*. This is why it has been of great utility in fermionic theoretical research the application of the exactly solvable Lipkin Model (LM) [2,3,4,5]. The LM greatly clears the way for assessing the validity and/or usefulness of distinct approaches formulated so as to research the manifold features of the fermion many body problem [6].

### 1.1. Quasi Spin Operators and Exactly Solvable Models

We will use such models in this work. The celebrated, two energy-levels Lipkin model (LM) (containing *N* fermions) is centered on an SU2 algebra. Such algebra is generated by operators called quasi-spin ones. We will here work with LM-variants that have immediately accessible exact analytical solutions (LM instead requires numerical diagonalization). Our analytical solutions can then be compared with results that emerge from variegated sorts of approximate theoretical N-fermions techniques. A relevant Casimir operator (CO) characterizes both the LM model [3] and our variants. The CO has attached to it different multiplets. Mostly the unperturbed ground state multiplet is the focus of attention [3]. Each level of our two energy ones is degenerate and accommodate Ω=N/2 fermions. We say that each of our two energy levels contains Ω sites in the upper level and the dame number in the lower level. Each site that can be occupied or empty. Sister sites are those in the same position above (higher energy level) and below (lower energy level).

Cambiaggio and Plastino (CP) [1,7,8,9] proposed a simple Lipkin-model extension, from SU2 to SU2 × SU2, to del with the excited Lipkin-multiplets (or bands) and thus adequately face the pairing interaction responsible for superconductivity. The augmentation allowed for the formulation, in quasi-spin parlance, of a BCS-like enactment which permits, as promised above, to exactly mimic superconductivity, yielding exact analytic solutions. In the CP-model (CPM), the BCS-solution coincides with the exact solution. The CPM is indeed an extension of the Lipkin model to a layout containing a variable particle number. Superconductivity quite often arises in fermion systems [10,11,12].

Colloquially, some physicist usually speak of phase transitions when the actually mean level-crossings. We will also do this here where we wish to detailed study, in a statistical order-disorder context, the combination of the pairing interaction with a spin-flip force, which can be done if we juxtapose the CP model of the pairing interaction with the Plastino-Moskowski (PM) model of a spin-flip interaction [13,14]. These two models display, individually, phase transitions(PT). We wish to see how these different PT mutually interfere. Our exactly solvable juxtaposition (CP and PM models) presents rich enough quantum structural features to profitably delineate such competition layout. Some preliminary details of the competition were reported in Refs. [8,9]. Here we present a more detailed view focused on and order-disorder scenario, discussing mainly the energy costs incurred in phase transitions, a colloquial vocable often used for level-crossings.

### 1.2. Goal: Our Route

Here we wish to construct the route detailed below and address the issues the this building up will generate.

- The central and well established idea is that fermion-fermion interactions are capable of generating phase transitions (PT) in many-fermions systems. The details of the PT depend on the properties of the extant fermion-fermion interaction.
- We wish to connect phase transitions (or level crossings in a finite system) with *changes* in the degree of statistical (CDSO) order exhibited by our many fermion system.
- We wish to analyze the fact that CDSO are made at the expense of energy spending.
- We wish to associate a new statistical quantifier to this energy expense.
- We wish to ascertain that this new quantifier is a very good phase transition “detector”.

## 2. Statistical Order, Disorder, and Disequilibrium

Our system is described by an unperturbed Hamiltonian $H_{0}$ plus two different interaction terms: a short range force (pairing) ($H_{CP}$) and a long range one (spin-flip) ($H_{M}$). There are attractor-states for each of them [8,9]. We wish to show that a kind of order is associated to these special states. We will undertake a canonical ensemble analysis of our system, whose protagonist is our Hamiltonian

(1) $$H=H_{0}+H_{M}+H_{CP}.$$

There exist two T=0-attractors. We assert that they are endowed with “order” in this respect: *every site is equally occupied*. For the *M* attractor, each sister-pair of sites is singly occupied. We find the same number of nucleons in each of the two energy-levels. This last feature is also displayed by the CO attractor, but now each sister-pair is either doubly occupied or empty. For us, “disorder” is associated to an instance in which each of the two energy-levels displays different occupation numbers. Using statistical tools we will attempt to the interplay between our two different interactions and see how they influence the order degree.

The Hamiltonian (1) represents a scenario that prevails in many atomic nuclei, in which a long-range distance interaction (e.g., a quadrupole one) competes with a short range one, the pairing interaction. *H* mimics such picture. Its (*H*) pairing component has identical mathematical properties as those of the actual pairing force. *H* also displays a spin-flip interaction.

### Statistical Order and Disequilibrium Quantifier D

That represented by *D* is a much employed concept (see for example Refs. [15,16,17,18,19,20,21]). Consider two opposite layouts: (i) perfect order or (ii) maximal randomness (no correlations at all) [15]. In between them variegated degrees of correlation may be found. In Ref. [15] a way such multiplicity was advanced. Since we are working in statistical fashion, with probability distributions (PD) as protagonists, we confidently state that maximum randomness or maximal disorder is represented by the uniform PD, The degree of order associated to a given PD is quantified by the distance of this PD (in probability space) to the uniform PD. This distance was called the disequilibrium *D*.

Great progress was made in Ref. [15] by proposing this measure *D* [16], that allow one to build up a sort of hierarchy. If there are privileged states among the accessible ones, their associated *D* value would reveal it. Multiplying *D* by the entropy *S* L. Ruiz, Mancini, and Calvet (LMC) [15] established one of today’s most used form for a statistical complexity *C*.

(2) C=DS,

a functional of the probability distributions (PDs) that competently lays hold of complexity in the fashion that entropy does so with randomness [15]. *D* adopt the form, if one deals with *N* accessible states [15,21],

(3) $$D=\sum_{i=1}^{N}\;{\left(p_{i}-\frac{1}{N}\right)}^{2}.$$

Here $\left\{p_{1},p_{2},\ldots ,p_{N}\right\}$ are the individual normalized probabilities ($\sum_{i=1}^{N}\;p_{i}=1$) [15]. *D* acquires the maximum possible value for a fully ordered state and instead vanishes in the case of uniform $p_{i}$. Of course, for the entropy we have $S=-\sum_{i=1}^{N}\;p_{i}\ln p_{i}$. LMC’s scheme attracted great attention (Refs. [15,16,17,18,19,20,21] constitute just a small sample) and was employed in variegated environments for both the canonical, micro-canonical, and grand canonical Gibbs’ ensembles.

## 3. Details of the Two Interactions We Are to Confront in This Work

### 3.1. Present Hamiltonians: (1) Unperturbed $H_{0}$, (2) Spin-Flip $H_{M}$, and (3) Pairing $H_{CP}$ Ones

One faces, as stated above

(4) $$H=H_{0}+H_{M}+H_{CP},$$

which is the juxtaposition of two distinct analytically solvable nucleon-nucleon interactions. Firstly, a spin-flip one advanced in Ref. [14] that represents either (1) spin-flip or, (2) forward scattering interactions [14]. The last term is the superconducting one [1,7] that mimics the pairing interactions that originates superconductivity [10]. The math background is that of the SU2 × SU2 group [8,9] involves *N* nucleons apportioned to 2Ω-fold degenerate single-particle (sp) levels (N=2Ω) separated by an energetic gap ϵ (we work here with ϵ-energy units). The system’s sp states are singled out by appeal to two quantum numbers: p,μ, with p=1,…,2Ω and μ=±1. *p* is called a quasi-spin quantum number and is regarded as a “site” [3].

### 3.2. Quasi-Spin Language and the Pairing Operators

The so called SU2 quasi-spin operators $J_{i}$ were introduced in Ref. [3] in terms of creation and destruction operators $C^{+},\;C$

(5) $$J_{z}=\left(1/2\right)\sum_{p,\mu }C_{p,\mu }^{+}C_{p,\mu },$$

(6) $$J_{+}=\sum_{p}C_{p,+}^{+}C_{p,-},$$

(7) $$J_{-}=\sum_{p}C_{p,-}^{+}C_{p,+}.$$

In Ref. [7] its authors introduced additional SU2 operators, angular momentum-like “pairing” operators

(8) $$Q_{0}=\left(1/2\right)\sum_{p,\mu }C_{p,\mu }^{+}C_{p,\mu }-\Omega^{+},$$

(9) $$Q_{+}=\sum_{p}C_{p,+}^{+}C_{p,-}^{+},$$

(10) $$Q_{-}=\sum_{p}C_{p,-}C_{p,+}.$$

It is clear that $Q_{+}$ generates and $Q_{-}$ destroys a pair of particles giving zero contribution to the $J_{z}$-value, or “coupled” to $J_{z}=0$. The ensuing coupled particles do not contribute to the total $J_{z}$ value. Any *J*-operator will commute with all *Q*-operators, and vice versa (SU2 × SU2).

### 3.3. Eigenvalues of *H*

Here, the pertinent, complete orthonormal basis is that of the eigenvalues of $J^{2},J_{z},Q^{2},Q_{0}$, with eigenstates $|J,Q,J_{z},Q_{0}\rangle$. Ref. [7] advanced an additional and quite useful quantum number denominated the quasi-spin seniority number ν

(11) ν=2(Ω−Q),

which tells the reader which is the number of “uncoupled” particles (not “paired" to $J_{z}=0$). Thus, ν is the number of “unpaired" particles in a *Q*-multiplet. As demonstrated in Ref. [7] we have

(12) J=ν/2,

(13) J+Q=Ω.

For the Lipkin model [3], N=2Ω, $Q_{0}=0$ [7], equalities that will be verified in our subsequent proceedings. The unperturbed ground state (ugs) is the eigenvalue of our unperturbed Hamiltonian $H_{0}$. For it one has $J=\Omega ,\;\;J_{z}=-\Omega ,\;\;Q=Q_{0}=0$ [7]. This state belongs to the multiplet $J=\Omega ,Q=Q_{0}=0,$ [see (15) below]. Returning to the spin-flip Hamiltonian $H_{PM}$ note that it reads [8,9]

(14) $$H_{PM}=H_{0}+H_{M}=H_{0}-V\left[J^{2}-J_{z}^{2}-N/2\right].$$

It is expressed via *J*, $J_{z}$, and a coupling constant *V*. Of course, if $J_{z}|J,J_{z}\rangle =M|J,J_{z}\rangle$, then $J^{2}|J,Q,J_{z},Q_{0}\rangle =M\left(M+1\right)|J,Q,J_{z},Q_{0}\rangle$. The pertinent eigen-states are denoted as $|J,Q,J_{z},Q_{0}\rangle$. Thus we see that

(15) $$H_{0}=J_{z}.$$

Naturally, the $H_{PM}$-eigenvalues become [14]

(16) $$E\left(J,Q,J_{z},Q_{0}\right)=Mz-V\left[M(M+1)-M2-N/2\right].$$

The energy of the unperturbed (V=0) gs (ugs) ($\nu =N,\;\;\;Q=Q_{0})$ reads

(17) $$E_{0}=-\Omega .$$

### 3.4. Phase Transitions or Level Crossings

The most important characteristic of $H_{PM}$ is that, as *V* augments the system unveils Ω phase-transitions (level crossings): the ground state (at T=0) stops being characterized by $J_{z}=-J$ and proceeds to be identified by successively larger $J_{z}$ values until one sets foot on M=0 at V=1 [14]. Abusing language a bit we say that the ket |J,M=0〉 is a kind of attractor for the system’s state if *V* is large enough (at T=0). This T=0-attractor (we call it I) is set apart by the feature that all quasi-spin sites *p* are occupied by a single fermion.

If we add now the pairing interaction to the Hamiltonian via the interaction term $\frac{G}{2}{\hat{Q}}_{+}{\hat{Q}}_{-}$. One has the the pairing Hamiltonian $H_{CP}$

(18) $$H_{CP}=H_{0}-\frac{G}{2}{\hat{Q}}_{+}{\hat{Q}}_{-},$$

which adds to $J_{z}$ a pairing contribution $E_{p}$

(19) $$E_{P}=-\left(G/2\right)Q\left(Q+1\right).$$

$H_{0}+H_{CP}$ exhibits its own phase transition (level crossing) at G=1 [7]. At such value the system turns out to be a superconductor [7]. $H_{CP}$, for large enough *G*, displays a second T=0-“attractor” II state, the superconducting-one, identified also by M=0 (as the one for $H_{PM}$) but in which half the sites display double -occupancy and the other half is empty.

## 4. Statistical Mechanics, Gibbs’ Canonical Ensemble, and *H*

We need first of all to recapitulate statistical tools introduced in Ref. [22]. For treating the ground state at T=0, we have to concern ourselves just with the J+Q=Ω “band". If T≠0 a manifold of states belonging to different bands are to be paid attention to. The associated degeneracy Y(J,Q) (computed in Ref. [22]) becomes, if β stands for the inverse temperature 1/T,

(20) $$Y\left(J,Q\right)=\frac{\left(2\Omega +2)!(2\Omega )!(2J+1)(2\Omega +1\right)}{(\Omega +J+Q+2)!(\Omega +J-Q+1)!(\Omega -J+Q+1)!(\Omega -J-Q)!}.$$

The partial partition function that runs only over *M*, let us call it $Z_{M}$, has the form [22]

(21) $$Z_{M}\left(\beta \right)=\sum_{M=-J}^{M=J}\;\exp\left\{-\beta \left[\left[M-V\left(J^{2}-M^{2}-J\right)\right]-\left(G/2\right)Q\left(Q+1\right)\right]\right\}.$$

while actual system’s true partition function *Z* reads

(22) $$Z\left(\beta \right)=\sum_{J,Q}\;Y\left(J,Q\right)Z_{M}\left(\beta \right),$$

where the quantum numbers *J* and *Q* run over all the values permitted by the SU2 × SU2 structure [22], that is:

- (23) 0≤J≤Ω,
- (24) 0≤Q≤Ω,
- (25) 0<J+Q≤Ω.

Of course, *Z* permits one to obtain all the thermodynamic information one might require.

Now we will slightly modify (25) in the fashion

(26) 0≤J+Q=s≤Ω.

So as to implement the *J*-*Q* sum we sum over J+Q=s and over *J*, with *Q* fixed at Q=s−J. We obey

(27) 0≤s≤Ω.

Note that s=0,1,2,3,…,Ω while J=0,1,2,.…,s. Finally, the partition function acquires the form

(28) $$Z=\sum_{J,Q}\;Y\left(J,Q\right)Z_{M}=\sum_{s=0}^{\Omega }\sum_{J=0}^{s}\;Y\left(J,Q\right)Z_{M}.$$

Following Ref. [3], we set N=2Ω. The level-energies are

(29) $$E\left(J,M,Q\right)=M-V\left(J^{2}-J-M^{2}\right)-GQ\left(Q+1\right)/2,$$

and define A(J,M,Q)=−βE(J,M,Q) so that our probabilities read

(30) $$P\left(J,M,Q\right)=\frac{Y(J,Q)\exp[A(J,M,Q)]}{Z},$$

leading to a mean energy *U*

(31) $$U=\sum_{J,Q}\sum_{M}P\left(J,M,Q\right)E\left(J,M,Q\right).$$

We are also interested in particular average energies like the mean pairing energy

(32) $$U_{P}=-\left(G/2\right)\sum_{J,Q}-P\left(J,0,Q\right)Q\left(Q+1\right),$$

and the mean spin-flip energy

(33) $$U_{M}=\sum_{J,Q}\sum_{M}P\left(J,M,Q\right)\left[M-V\left(J^{2}-J-M^{2}\right)\right],$$

Also, the free pairing energy

(34) $$F_{P}=U_{P}-TS,$$

and the the free spin-flip energy

(35) $$F_{M}=U_{M}-TS.$$

Remind that maximal randomness or total disorder is associated to the uniform distribution. In our context we have in such a case

(36) $$P_{unif}=\frac{1}{V_{u}},$$

where $V_{u}$ is here given by the binomial value for 4Ω/N. Our present and all important disequilibrium *D* becomes

(37) $$D=\sum_{J,Q}\;\sum_{M=-J}^{M=J}{\left[P\left(M,Q\right)-P_{unif}\right]}^{2}.$$

For the entropy we find

(38) $$S=-\sum_{J,Q}\;\sum_{M=-J}^{M=J}P\left(M,Q\right)\ln P(M,Q).$$

### Scheme of Our Algorithm

1. Since our models are analytically solvable, we know the expressions for the exact energies $E_{i}$. We also know the temperature *T*.
2. Accordingly, we can construct our probability distribution (PD), whose elements $P_{i}$ are $\propto \exp\left[-\beta E_{i}\right]$, with β the inverse temperature
3. Since we have the $P_{i}$ we can compute the entropy $S=-\sum_{i}\;P_{i}\ln P_{i}$ and the mean energy *U*, $U=\sum_{i}\;P_{i}E_{i}$.
4. With these quantities we compute the free energy F=U−TS.
5. We also know the disequilibrium *D*, which is a simple function of the $P_{i}$, given above in Equation (3).

## 5. Information Cost (in Free Energy), a New Statistical Quantifier

We pass now to describe the main innovation of this paper, a new statistical quantifier related to the disequilibrium *D*. We contemplate in this scenario two control parameters $V\equiv X_{1}$ and $G\equiv X_{2}$. A perturbation in the control parameter, let us say from *V* to V+dV, will result in a change of the system’s associated degree of order (DOO). Inspired by Ref. [23], we introduce the order-efficiency ν of our interactions in the fashion

(39) $$\nu \left(X;dX\right)=k_{B}\frac{dD}{dW},$$

with $k_{B}$ Boltzmann’s constant. Our dD and dW are, respectively, the variations in disequilibrium and the work done on the system as a result of the dX change. Thus, ν(X;dX) represents the diminution (increase) in uncertainty (for our system’ state) that results from each unit of work done on the system. A small value of ν indicates that much work on the system is needed to modify the current order degree. Vice versa in the case of large ν. Of course, if dF is negative, it is the system itself that does the pertinent work. Such will be the case below. In quasi-static processes, for which we things happen slowly enough that the system effectively adjusts instantaneously to a new equilibrium state, it can be demonstrated following the parallel treatment given in Ref. [23] (for a different purpose) that

(40) $$\nu \left(X;dX\right)=k_{B}\frac{\partial D}{\partial F}=\frac{\partial D}{\partial X}/\frac{\partial F}{\partial X},$$

involving Helmholtz’ free energy *F*. *X* stands for either of our two coupling constants. We will always set $k_{B}=1$. The derivatives can be performed analytically. The modification in *F* can be associated to the work done on the system dF=dW [23]. On the other hand, we may regard $\nu_{X}$ as the work required (in varying *X*) so as to increment (ν<0) or diminish (ν>0) our information concerning the system. This represents an “information cost”. Alternatively, $\nu_{X}$ is the work needed to augment (decease) the degree of order in the system

Let us emphasize that

- If dD>0 we see increasing statistical order,
- If dD<0 we see increasing statistical disorder.

### The Conjugate Extensive Counterpart of ν

Consider now the extensive quantity

(41) f=−dF/dX,

where the intensive variable *X* stands for either *G* or *V*. Since the entropy *S* is

(42) S=−dF/dT,

*f* would be a sort of “quasi-entropic” counterpart of *X*. We will ascertain below whether this counterpart has something useful to say about our present endeavor. We will call *f* the information cost of changing the X− value. Instead, −f signals an information “source”, a kind of negentropy that tells us which is the amount of energy involved in varying *D* when *X* changes. As always, negentropy is a measure of order (and so is our quasi-negentropy). The notion of negative entropy was advanced by Schrödinger in the book *What is Life?* [24]. Later, in 1974, Brillouin baptized the notion as negentropy [25]. Thus, we can associate −f to the extension of this notion from *S* to *D*.

## 6. Results Obtained with Our New Quantifier ν at Finite Temperature *T*

### 6.1. ν Detects the Superconductivity Transition: $\nu_{G}$ versus G Plots

**Figure 1:**
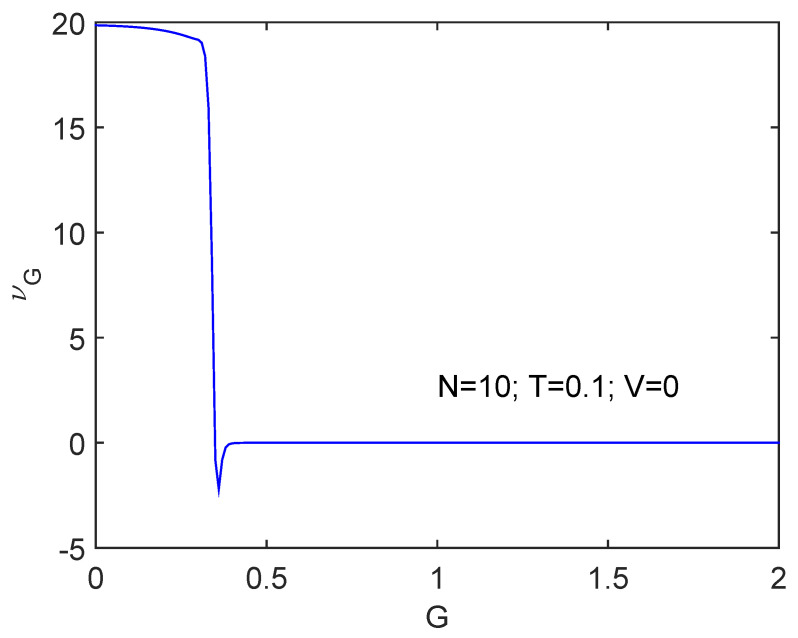
We depict $\nu_{G}$ versus *G* for V=0, N=10, and T=0.1 (units for which $k_{B}=1$). There is a significant drop at the critical G-value at which superconductivity becomes established. ν clearly detects the phase transition.

We will work at T=0.1 (units for which $k_{B}=1$). Figure 1 displays $\nu_{G}$ versus *G* for N=10. At a critical value of the pairing constant the phase transition occurs and is duly detected by ν. Note that ν is positive before the transition to superconductivity and slightly negative when the later becomes established. This entails, as expected, that the physics has radically changed. Of course, the critical *G* value $G_{crit}$ is slightly varied from its T=0 value 0.3333 because of finite temperature effects.

**Figure 2:**
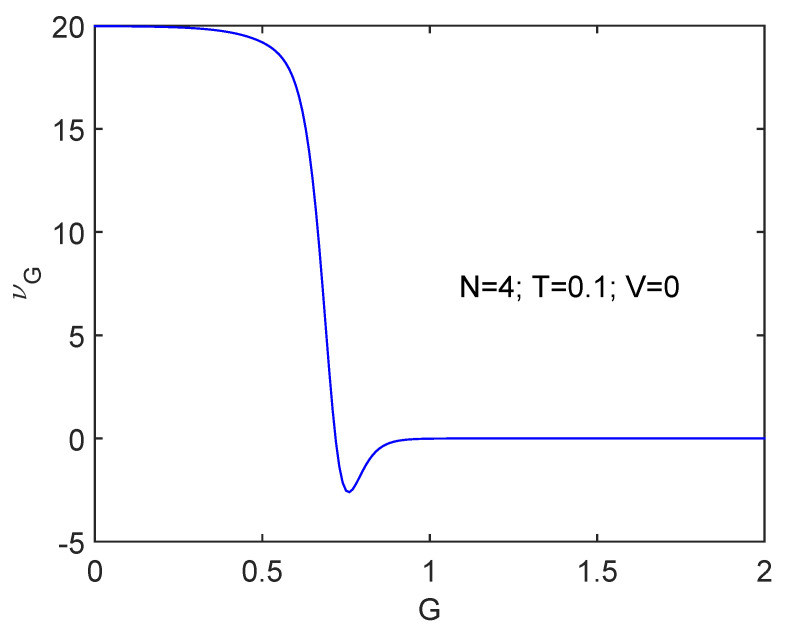
Same as Figure 1 but for N=4. Things proceed in a rather smoother fashion for N=4 that for N=10.

Figure 2 displays results for the same scenario but for N=4, where $G_{crit}=2/3$ at T=0.

The transition is more clearly seen as *N* augments.

### 6.2. ν Detects the Spin-Flip Transition: $\nu_{V}$ versus V Plots

**Figure 3:**
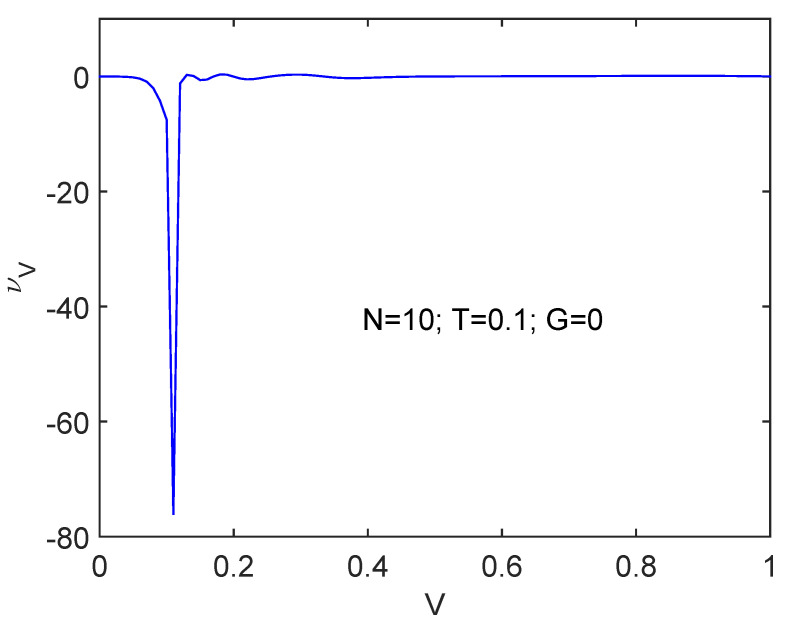
Spin-flip interaction workings at T=0.1, G=0, and N=10. We depict $\nu_{V}$ versus *V*. At the critical spin-flip coupling constant $V_{crit}$ order suddenly augments, but after the transition, things return to the original stage as *V* keeps growing.

**Figure 4:**
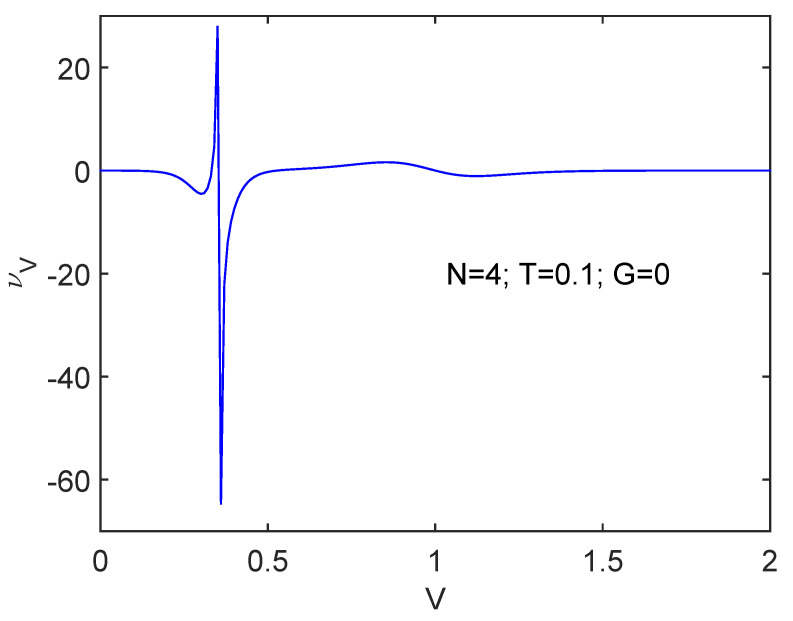
Same as Figure 3 for N=4. The transition region exhibits a much sharper definition though.

We pass now to consider the spin-flip interaction in Figure 3 at T=0.1, N=10, and G=0. Immediately we realize that a totally different scenario is being confronted. At the critical *V* value $\nu_{V}$ suddenly changes but its precedent value is re-established after the transition while *V* keeps growing. However, there emerges here a much stronger *N*-dependence than that for the pairing force, as illustrated by Figure 4 for N=4. Here the aspect of things at the phase transition looks different in the two N=4,10 systems. $\nu_{V}$ first augments for N=4 and immediately after wards decreases. Opposite to the pairing case above, here the phase transition is displayed in a much more clear-cut fashion smaller *N*’s than for larger ones.

### 6.3. The Statistical Extensive Measure F

**Figure 5:**
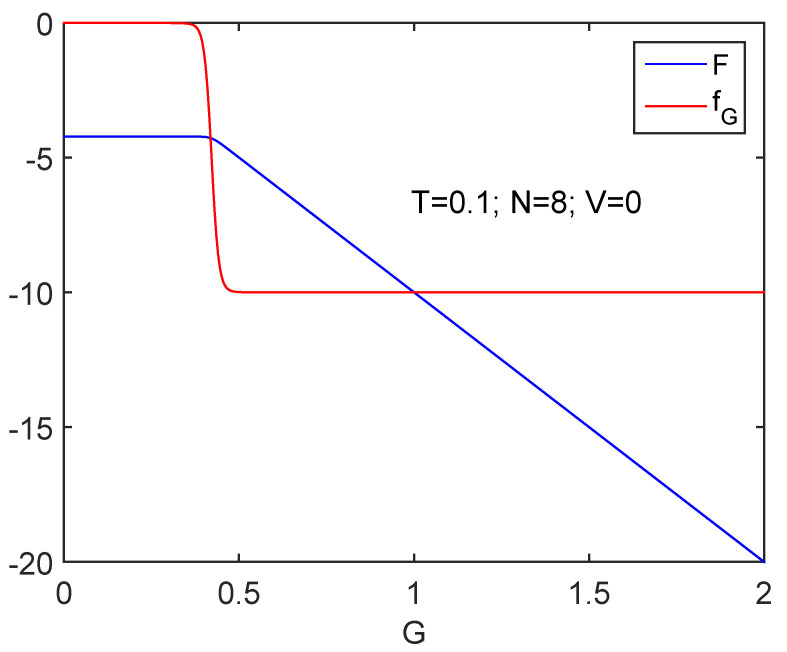
Information cost $f_{G}$ for N=8 clearly detects the phase transition. As one should expect, at $G_{crit}$ order grows (see text). The free energy *F* is also displayed. As *G* augments, work is done BY the system.

**Figure 6:**
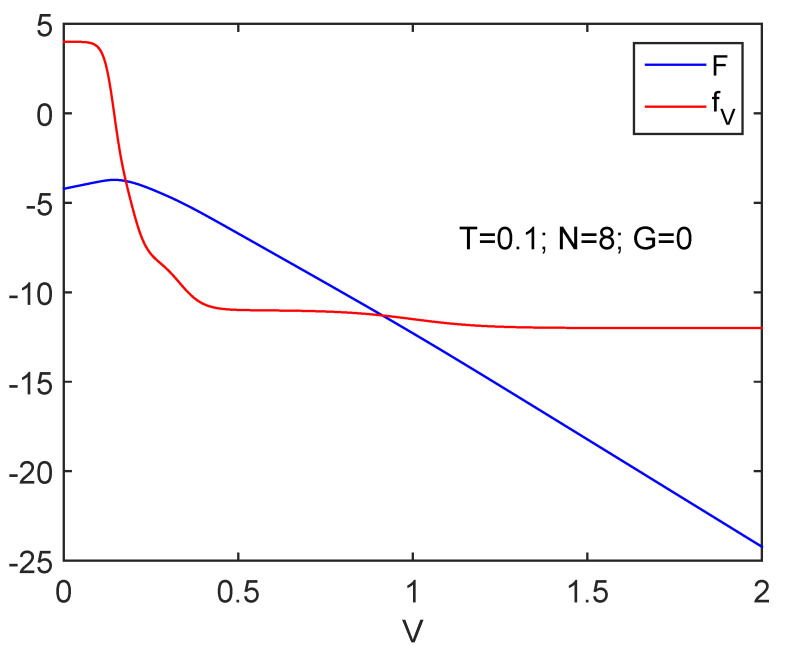
Information cost $f_{V}$ (N=8) detects the spin-flip transition. The free energy *F* is also displayed. As *V* augments, work is done BY the system as *V* grows.

We start discussing the information cost *f* in Figure 1, where we depict $f_{G}$ versus *G* together with the free energy *F*. The behavior of *F* is of critical importance to decide whether we speak of order or of disorder. We know that ν decreases at the superconductivity transition. We also see in the graph that dF is negative. Thus, dD>0 and order augments. Figure 5 and Figure 6 show that the quasi-negentropy grows at the phase transition.

Figure 6 is the counterpart of Figure 1 for $f_{V}$. Following the reasoning line found at the start of this Subsection, we ascertain that order grows.

### 6.4. Three Dimensional Graphs

**Figure 7:**
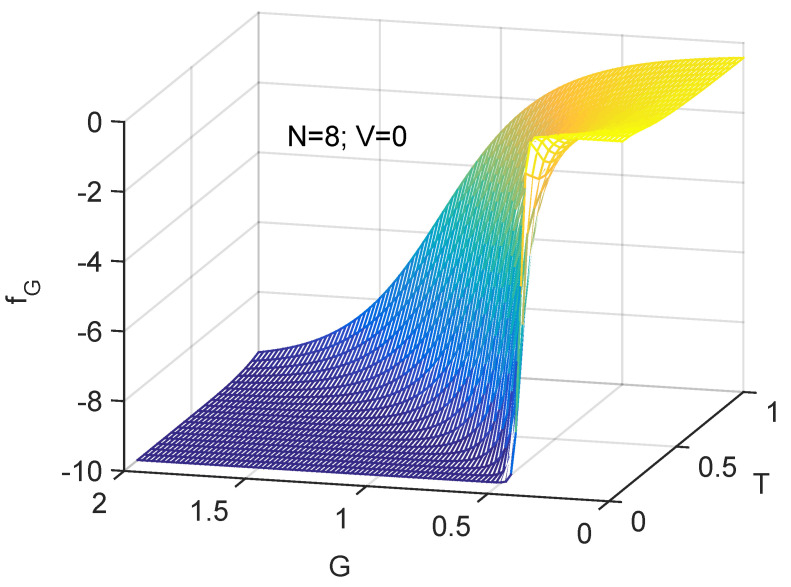
Three dimensional plot. $f_{G}$ as a function of both *T* and *G*. One notes the competition between the two intensive quantities *T* and *G*. Clearly, statistical order grows with *G* and diminishes with *T* as one should expect.

**Figure 8:**
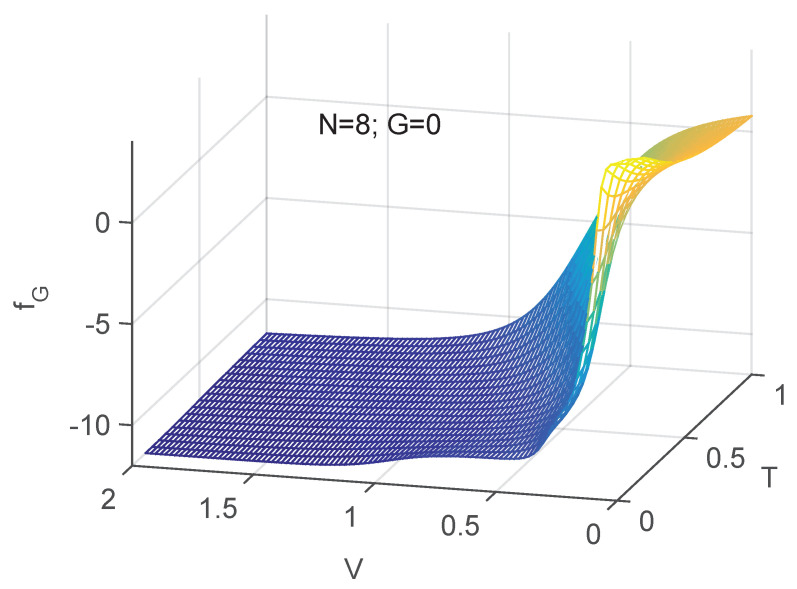
Three dimensional plot. $f_{V}$ as a function of both *T* and *V*. One notes the competition between the two intensive quantities. Clearly, order grows with *V* and diminishes with *T* as one should expect.

A more transparent illustration of the physics of order here analyzed is that provided by Figure 7 and Figure 8. The statistical disorder-induced role of the temperature *T* is clearly visible.

## 7. Conclusions

In this work we have revisited the interplay statistical order-disorder, using the lens of a new statistical quantifier ν (and its conjugate $f_{X}$) that are a measure of energetic cost (in terms of free energy) of changes suffered by the order-disorder quantifier called the disequilibrium *D*.

If we change the sign of the extensive counterpart of ν called $f_{X}$ (the coupling constant *X* being either *G* or *V*), we have a quasi-negentropy. This brings together the notions of order and energy expense. We have studied the behavior of ν with regards to two competing interactions. These interactions are a spin-flip and a short-range ones Our layout is discussed using an exactly solvable model of the Lipkin-sort, that permits one to undertake exact treatments of the pertinent Hamiltonians.

We contrast the statistical order-disorder properties of these two kinds of interaction. They are indeed different, in particular in relation to their response to increases in temperature.

Thus, we can reasonably claim to have unveiled a new sort of differences, in order and energy cost, between pairing (short range) and multipole (long range) interactions.
